# Stability and assembly *in vitro *of bacteriophage PP7 virus-like particles

**DOI:** 10.1186/1477-3155-5-10

**Published:** 2007-11-26

**Authors:** Jerri C Caldeira, David S Peabody

**Affiliations:** 1 Department of Molecular Genetics and Microbiology, University of New Mexico School of Medicine, Albuquerque, NM87131, USA

## Abstract

**Background:**

The stability of a virus-like particle (VLP) is an important consideration for its use in nanobiotechnology. The icosahedral capsid of the RNA bacteriophage PP7 is cross-linked by disulfide bonds between coat protein dimers at its 5-fold and quasi-6-fold symmetry axes. This work determined the effects of these disulfides on the VLP's thermal stability.

**Results:**

Measurements of the thermal denaturation behavior of PP7 VLPs in the presence and absence of a reducing agent show that disulfide cross-links substantially stabilize them against thermal denaturation. Although dimers in the capsid are linked to one another by disulfides, the two subunits of dimers themselves are held together only by non-covalent interactions. In an effort to confer even greater stability a new cross-link was introduced by genetically fusing two coat protein monomers, thus producing a "single-chain dimer" that assembles normally into a completely cross-linked VLP. However, subunit fusion failed to increase the thermal stability of the particles, even though it stabilized the isolated dimer. As a step toward gaining control of the internal composition of the capsid, conditions that promote the assembly of PP7 coat protein dimers into virus-like particles *in vitro *were established.

**Conclusion:**

The presence of inter-dimer disulfide bonds greatly stabilizes the PP7 virus-like particle against thermal denaturation. Covalently cross-linking the subunits of the dimers themselves by genetically fusing them through a dipeptide linker sequence, offers no further stabilization of the VLP, although it does stabilize the dimer. PP7 capsids readily assemble *in vitro *in a reaction that requires RNA.

## Background

Viruses and VLPs are currently under investigation for a variety of uses that include confinement of chemical reactions, as templates for materials synthesis, as molecular electronics components, as platforms for polyvalent display of antigens and other ligands, and for targeted drug delivery. For some relevant examples see references [1-13]. The single-strand RNA bacteriophages offer certain advantages for such applications. VLPs can be produced in large quantities by self-assembly of a single coat protein polypeptide expressed from a plasmid, thus allowing extensive genetic manipulation of the capsid without the constraints imposed by the necessity to maintain virus viability [14]. Engineering is further facilitated by detailed knowledge of the three-dimensional structures of RNA phages [15-22].

The physical stability of a VLP is clearly one of the factors that influence its suitability for a given application. The capsids of certain RNA phages are cross-linked by disulfide bonds between coat protein dimers at the five-fold and quasi six-fold symmetry axes, and these cross-links are expected to stabilize the capsid. It is well known that naturally occurring disulfide bonds generally stabilize protein structure (see ref [23], for example). The experiments reported here confirm this expectation for VLPs of the *Pseudomonas *RNA phage PP7.

## Results and discussion

### PP7's disulfide bonds stabilize the capsid

Capsid stability was assessed by measuring the quantity of intact VLP and soluble protein remaining after incubation at different temperatures (see Materials and Methods for details). Briefly, samples of purified PP7 VLPs were heated in a PCR thermocycler in either the presence or absence of dithiothreotol (DTT), and, after two minutes, the samples were chilled on ice and subjected to centrifugation at 13,000 rpm in a microcentrifuge. The pellet and supernatant were designated as insoluble and soluble fractions respectively and the amount of protein in each was determined by the assay of Bradford [24]. The soluble fraction was also analyzed by agarose gel electrophoresis under native conditions where virus-like particles have a characteristic mobility. After staining, the quantity of capsids surviving heat treatment was determined by densitometry.

**Figure 1:**
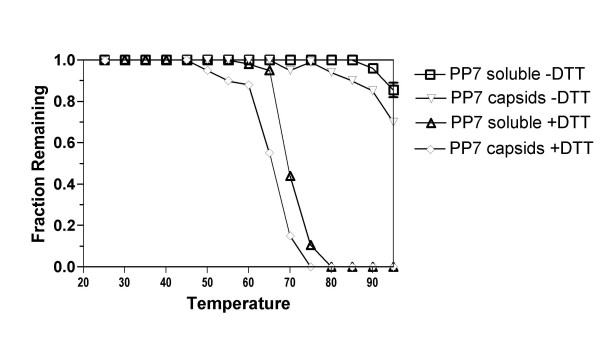
The stability of PP7 virus-like particles under reducing (+DTT) and under non-reducing (-DTT) conditions as measured by the fraction of capsids or soluble coat protein remaining after heating for two minutes at the indicated temperatures.

Figure 1 shows that PP7 VLPs only began to denature at temperatures approaching 90°C. After two minutes at 95°C, the highest temperature tested, about 70% of capsids remained intact. A roughly equivalent fraction of PP7 coat protein (about 80%) remained soluble. However, the presence of DTT substantially reduced particle stability. Measurements of both VLPs and soluble protein showed sharp declines beginning at about 60 and 65°C. The thermal denaturation behavior of reduced PP7 is similar to that of MS2, a related RNA bacteriophage naturally lacking disulfide bonds (unpublished results).

**Figure 2:**
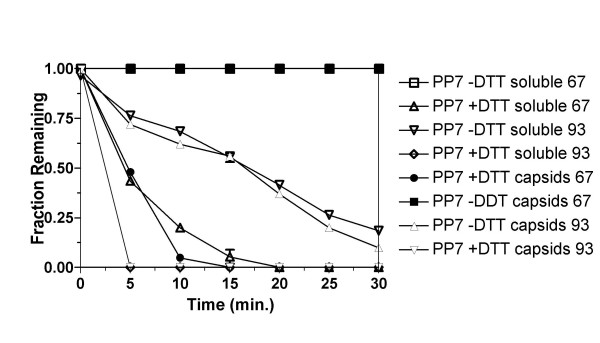
The stability of PP7 virus-like particles as indicated by the fraction of capsid or soluble protein remaining after heating for the indicated times at 93°C under non-reducing conditions, or at 67°C under reducing conditions.

The stabilizing influence of the disulfide bonds is also apparent in the rate of denaturation (Figure 2). Virus-like particles were heated at 67°C or at 93°C under reducing (+DTT) and non-reducing (-DTT) conditions, samples were removed at time intervals and subjected to measurements of intact VLPs and soluble protein as described above. These temperatures were chosen because they fall roughly within the reducing and nonreducing melting transitions in Figure 1. With disulfides intact (no DTT) VLPs were stable at 67°C over the 30-minute time course of the experiment, but in the presence of DTT both VLPs and soluble protein disappeared from the soluble phase with a half-life of about 5 minutes at 67°C. When heated at 93°C, unreduced VLPs and soluble protein disappeared with half-lives on the order of 15 minutes. but in DTT's presence 100% of capsids and soluble protein had already disappeared at 5 minutes, the earliest time point taken.

It should be noted that in each case the results obtained by following the movement of coat protein from the soluble to the insoluble fraction are similar to those obtained by measuring the disappearance of capsids, thus indicating that when capsids disaggregate, the individual coat protein subunits mostly denature concomitantly to an aggregated, insoluble form.

These results show that PP7 VLPs are substantially stabilized by the presence of its disulfide bonds. This is consistent with the well-known effects of naturally occurring disulfide bonds in many different proteins [23], and with the enhanced stability of bacteriophage MS2 VLPs resulting from disulfide bonds introduced at its 5-fold symmetry axes by genetic modification [25].

### Effects of fusing subunits of the coat protein dimer

Although disulfides cross-link coat protein dimers to one another in the PP7 capsid, there exists no cross-link between the two subunits of the dimer itself. Thus, pentamers and hexamers should be the largest covalent oligomers encountered when VLPs are denatured. However, adding a covalent cross-link between the two subunits of the coat protein dimer would join all 180 subunits of the capsid into a single, giant covalent molecule with a molecular weight of about 2.5 million. Would the presence of such an additional cross-link further increase capsid stability?

The proximity within the dimer of the N-terminus of one subunit to the C-terminus of the other suggested a simple means of introducing an inter-dimer covalent bond. Duplicating the coat gene and joining the two copies together in a single reading frame fuses the C-terminus of one monomer to the N-terminus of the other. Similarly constructed single-chain dimers of MS2 coat protein have been well characterized. They retain the functional characteristics of the wild-type protein; that is, they repress translation from the replicase translational operator and assemble into apparently normal VLPs. Moreover, the tethering of MS2 coat monomers to one another greatly stabilizes the dimer against chemical denaturation and frequently reverses the destabilizing effects of amino acid substitutions and peptide insertions.

**Figure 3:**
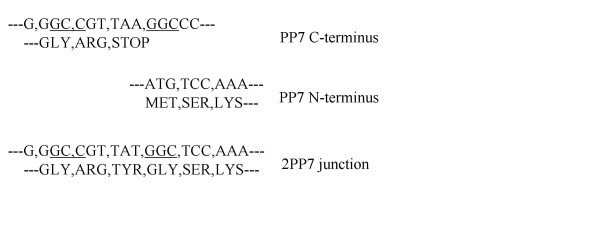
The sequence at the junction of the 2PP7 duplication is shown at bottom, and the sequences of the 5'- and 3' ends of the PP7 coat sequence are shown above it. In the 2PP7 construct the C-terminal arginine of the upstream copy is fused through a tyr-gly linker to the serine, residue number 2, of the downstream copy.

The single-chain PP7 dimer (we call it 2PP7) was constructed as described in Materials and Methods and contains the junction sequence shown in Figure 3. A gly-tyr dipeptide serves as a linker between the C-terminal arginine of the upstream coat sequence and the first amino acid (serine) of the downstream sequence. The presence of this particular junction was the consequence of the strategy for fusion of the duplicated sequence, which took advantage of a natural *Bgl *I site near the 3'-end of the PP7 coding sequence by joining it to a new *Bgl I *site introduced at the 5'-end of the downstream copy of the coat sequence. We do not know whether this is the optimal arrangement of linker length and sequence, but functional tests [26] indicate that the 2PP7 molecule is fully active in the repression of translation from the PP7 replicase translation initiation site (not shown). Moreover, it assembled into apparently normal capsids as evidenced by its behavior upon electrophoresis in agarose gels, where it produced a band with mobility similar to that of the normal PP7 VLP, and in columns of Sepharose CL4B, where it elutes in the same position as authentic PP7 VLPs (results not shown).

**Figure 4:**
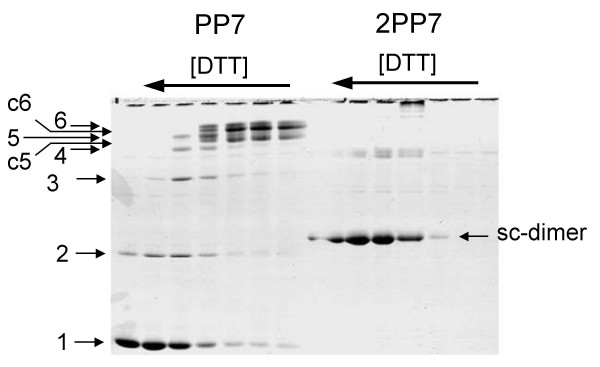
SDS polyacrylamide gel electrophoresis of the products of reduction of PP7 and 2PP7 VLPs with varying concentrations of DTT. Note that PP7 pentamers and hexamers each occur in two electrophoretic forms. Before reduction most pentamers and hexamers are circularly cross-linked around the 5-fold and quasi-6-fold viral axes. We call these c5-mers and c6-mers. Breaking one of the disulfides in the ring leads to linear pentamers and hexamers.

SDS-polyacrylamide gel electrophoresis of the products of partial reduction of PP7 and 2PP7 virus-like particles confirmed that the 2PP7 capsid was cross-linked into a covalent structure of high order (Figure 4). The unreduced PP7 particle yields two closely spaced main bands that likely correspond to circular pentamers and hexamers, and two less intense bands representing linear pentamers and hexamers (i.e. "nicked" circles). This is in accordance with the known arrangement of disulfide bonds in the PP7 particle [21], assuming that occasionally a disulfide bond is broken. The relative intensities of these species are consistent with the presence of 20 hexamers and 12 pentamers predicted from the structure of the icosahedron. Partial reduction of the PP7 VLP results in the appearance of monomers, and multiples of monomers up to the size of hexamers. However, VLPs made of single-chain dimers behave differently. Complete reduction of 2PP7 produces a single band at a position corresponding to twice the molecular weight of the wild-type monomer (i.e. the weight of the single-chain dimer), while unreduced material apparently fails to enter the gel. Partial reduction produces species that are apparently multiples of the single-chain dimer, but most of these products fail to penetrate the gel.

### Thermal stability of 2PP7 virus-like particles

**Figure 5:**
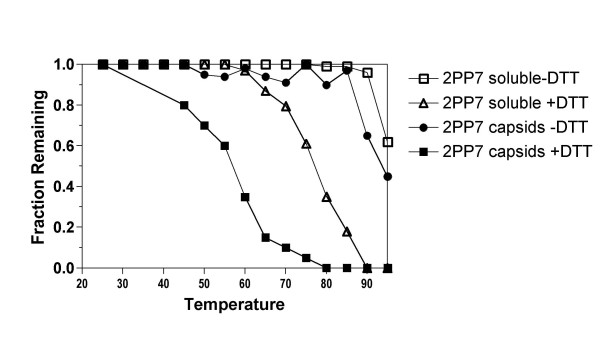
Stability of 2PP7 as a function of temperature. Samples were heated for two minutes at the indicated temperatures under reducing or non-reducing conditions and fractions of soluble protein and capsids remaining were determined.

Denaturation of 2PP7 virus-like particles after two minutes at a variety of temperatures is shown in Figure 5. With disulfide bonds intact the particle was stable up to a temperature of about 85–90°C. At the highest temperature tested (95°C) about 40% of VLPs remained, and about 60% of the protein was found in the soluble fraction. When DTT was added, the particles denatured at significantly lower temperatures, but just how much lower depended on whether VLPs or soluble protein was being measured. About half of VLPs disappeared in two minutes at around 55–60°C, but approximately half insolubility was not achieved until nearly 80°C. Note that 2PP7 capsids are actually a little less stable than PP7 VLPs. This may be due to crowding at the local 3-fold axes caused by the extra sequences present at the 2PP7 fusion junction (Figure 3).

**Figure 6:**
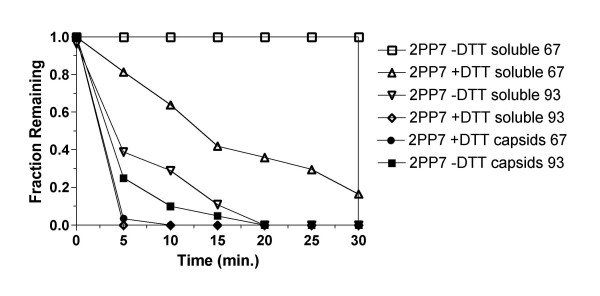
Disappearance of 2PP7 capsids and soluble protein with time when heated at 67°C under reducing conditions or at 93°C under non-reducing conditions.

These observations were echoed in the rates of disappearance of VLPs and soluble protein. Figure 6 shows how they declined as a function of time at 93°C with the disulfides intact. In this case, both measurements gave the same result; capsids and soluble protein disappeared more or less together a half-life on the order of only a few minutes. In contrast, when heated at 67°C in the presence of DTT the rate of capsid disappearance was far more rapid than the decline of soluble protein. What might explain this behavior?

Observations of the increased stability of single-chain dimers of MS2 coat protein have already been reported. The MS2 single-chain dimer is more stable than wild-type to urea denaturation [27] and is more resistant to the destabilizing effects of a variety of amino acid substitutions and peptide insertions [27-29]. It was reasonable to assume that a similar stabilization would occur in the case of PP7 single-chain dimers. The fact that subunit fusion failed to additionally stabilize the VLP suggests that the stability of the dimer is not a limiting factor in the stability of the capsid. In the case of the disulfide cross-linked 2PP7 particle, VLP disappearance and protein entry into the insoluble fraction are linked events; the 2PP7 capsid is a single covalent molecule and denatures as a unit. However, when the disulfides are reduced, high temperature may cause VLP disassembly without concomitant subunit denaturation. Instead, elevation of temperature may first liberate single-chain dimers. which are substantially more stable than unfused (i.e. wild-type) dimers. Their irreversible denaturation, which is monitored by entry of the polypeptide into the insoluble fraction, occurs only at higher temperatures, thus accounting for the disparity between measurements of capsid and soluble protein loss. Alternatively, at temperatures where capsid disassembly is induced, single-chain dimers might first enter a reversibly denatured state, with irreversible denaturation and aggregation occurring only at still higher temperatures.

**Figure 7:**
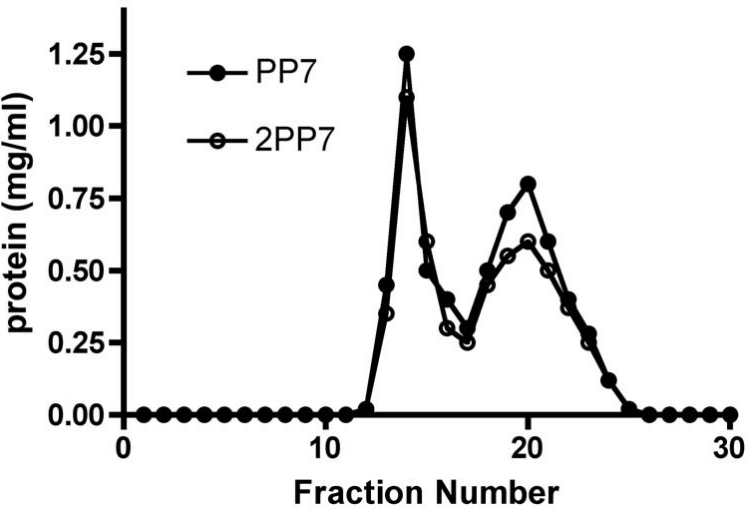
Purification of DTT/urea-disaggregated PP7 and 2PP7 coat protein by gel filtration chromatography in 10 mM acetic acid, 50 mM NaCl (about pH 4). To estimate the molecular weight of the putative dimer species, bovine serum albumin (68 kD), ovalbumin (43 kD), chymotrypsinogen (25.7 kD) and hen lysozyme (14.4 kD) were used as standards. They peaked at fractions 14, 18, 22 and 26 respectively.

In order to compare their stabilities, PP7 dimers and 2PP7 single-chain "dimers" were purified. Briefly, VLPs were denatured in 6 M urea in the presence of 10 mM DTT, at 0°C and then dialyzed against 10 mM acetic acid, 50 mM NaCl (about pH 4). Under these conditions the denatured coat protein refolds to the dimer, but its further assembly into the VLP is inhibited. This behavior is well known for other coat proteins and seems to hold for wild-type PP7 and 2PP7 as well. Figure 7 shows their identical elution profiles from Sephadex G75. Following a peak of aggregated material eluting in the void volume, a species appeared with a peak at fraction 20 and an apparent molecular weight (compared to standards) of about 32,000, a value that agrees reasonably closely with the predicted size of the dimer (about 28 Kd). Agarose gel electrophoresis shows that the material in the void volume is made up of capsids that failed to denature under these conditions. It should be noted that although both intact capsid and presumed dimer species are present in the column, they apparently do not equilibrate with one another under these conditions; electrophoresis conducted many days after chromatography shows that dimers do not generate VLPs in this buffer even on this time scale. We also note parenthetically that in experiments conducted more recently we observed that VLPs can be completely disassembled when denatured for a longer time (2 hours) at a higher urea concentration (8 M) and a higher temperature (37°C). When PP7 VLPs were denatured under these more drastic conditions and then renatured by dialysis into 10 mM acetic acid, 50 mM NaCl, only the dimer peak appeared in the column (results not shown).

**Figure 8:**
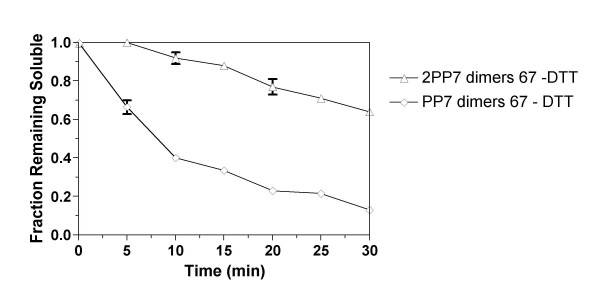
Stabilities of PP7 and 2PP7 dimers indicated by the disappearance of protein from the soluble fraction as a function of time at 67°C.

In Figure 8 the rates of disappearance of soluble forms of the two coat proteins when heated at 67°C are shown. Clearly the fused dimer was substantially more stable. After 30 minutes at 67°C more than 60% of 2PP7 dimers were still soluble whereas only about 20% of the wild-type protein remained soluble after 30 minutes. Surprisingly, free 2PP7 dimers were apparently more stable than the 2PP7 protein present in DTT-reduced capsids, since they exhibited markedly slower rates of appearance in the insoluble fraction (compare Figures 6 and 8). We do not know how to explain this difference.

### Assembly of PP7-like particles *in vitro*

**Figure 9:**
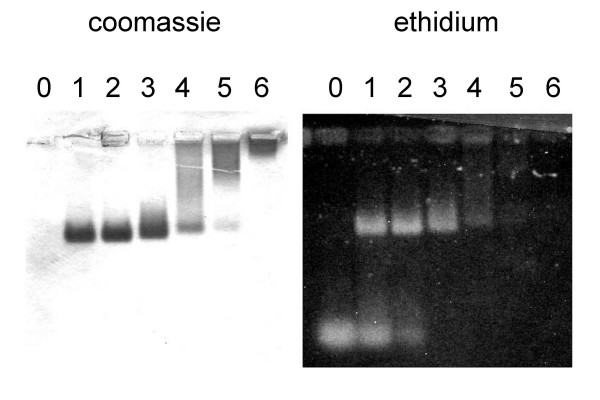
Assembly of PP7 VLPs *in vitro *in the presence of MS2 operator RNA. All reactions contained 0.1 nmol PP7 dimers and MS2 translational operator RNA in amounts varying from 0.1 nmol in lane 1 (by 2-fold serial dilutions) to 6.3 pmol in number 5. Lane 0 is 0.1 nmol RNA without protein and lane 6 is 0.1 nmol protein without RNA.

**Figure 10:**
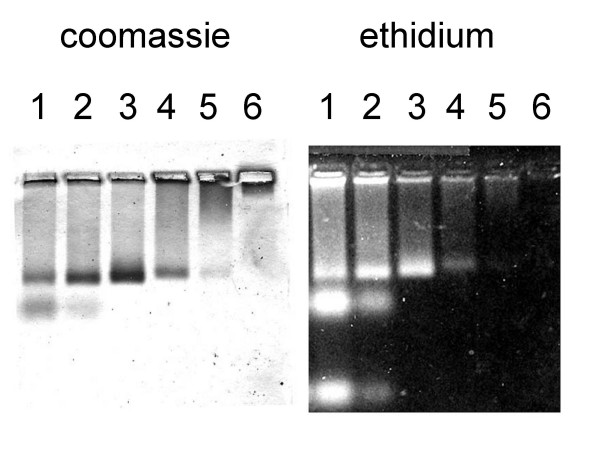
Assembly in the presence of PP7 translational operator RNA. Reactions were conducted at the protein and RNA concentrations given in the legend to Figure 9.

To assemble virus-like particles *in vitro*, the purified dimeric protein was added to reactions containing 50 mM Tris-HCl, pH 8.5 with varying concentrations yeast tRNA, bacteriophage MS2 translational operator RNA, or PP7 translational operator RNA. The amounts of protein and RNA in the reactions are given in the legend to Figure 9. After 30 minutes, the samples were subjected to agarose gel electrophoresis. RNA was visualized by staining the gels with ethidium bromide followed by photography on a UV transilluminator. Protein was detected by staining with Coomassie Brilliant Blue R250. The results obtained for assembly reactions carried out with MS2 operator RNA (Figure 9) were essentially identical to those with yeast tRNA (not shown). These non-PP7 RNAs induced the formation of particles that comigrated with VLPs purified from *E. coli*. Thus, although assembly requires RNA, it does not depend specifically on PP7 RNA. However, when assembly was conducted at high concentrations of PP7 operator RNA an additional electrophoretic species was formed that ran a little faster than the virus-like particle (Figure 10). This species contained both RNA and protein, because it stained both with ethidium bromide and coomassie blue. Preliminary quantitation of the RNA (in this case ^32^P-labeled) and protein (by comparison to a dilution series of PP7 virus-like particles at known concentrations) showed that the ratio of RNA to coat protein dimer is about 0.9, suggesting this species represents a one-to-one complex of unassembled coat protein dimer and PP7 operator RNA. High concentrations of operator RNA apparently inhibited virus assembly, since the one-to-one complex was most abundant at the highest RNA-to-protein ratio, and its quantity decreased as the RNA concentration was lowered. Meanwhile, as the RNA-to-protein ratio decreased, the yield of capsids first increased and then diminished again as the RNA concentration fell below that required to promote full assembly. This inhibitory effect of RNA at high concentration is apparently specific to authentic PP7 operator RNA, since neither the MS2 operator nor tRNA seems to exert this effect.

Preliminary measurements of the amount of radioactive PP7 RNA present in VLPs at the highest RNA concentrations suggested an RNA to coat protein dimer ratio of about 0.3. In other words, since each dimer contains a single RNA-binding site, only about a third of the ninety sites present in the capsid were actually occupied. Apparently, the capsid is unable to enclose the quantity of RNA required to fully saturate the 90 RNA-binding sites present on its inner surface. With a length of 45 nucleotides, the RNA used in these studies was substantially larger that the minimum size (no more than 28 nucleotides) required for tight binding to PP7 coat protein. The PP7 genome itself is about 3,600 nucleotides long, so the incorporation of thirty 45-mers would not exceed the presumed packaging limit of the capsid. However, the interaction of coat protein with the translational operator concentrates the RNA at the inner surface of the capsid shell where intermolecular RNA-RNA crowding might prevent higher occupancy levels. Such crowding could also account for the relative inhibition of capsid assembly observed at high operator concentrations. However, results obtained recently with the related bacteriophage MS2 suggest an additional possibility: Binding of operator RNA may induce a coat protein dimer to adopt a conformation competent to initiate, but not to efficiently propagate capsid assembly. In other words, binding of operator RNA may be necessary to put the dimer in a state that is active for nucleation of assembly, but further addition of dimers requires that some of them be present in an RNA-free conformation [30]. Thus the presence of excess operator RNA is inhibitory of assembly.

## Conclusion

It is well known that naturally occurring disulfide bonds generally contribute to protein stability. The observations presented here show that the presence of disulfide bonds between coat protein dimers greatly stabilizes the PP7 virus-like particle against thermal denaturation. We sought to confer additional stability by genetically fusing the two subunits of the dimer. By thus creating a covalent cross-link between coat protein monomers, all 180 polypeptides of the VLP become cross-linked, either by disulfide bonds or by the subunit fusion. Although this manipulation stabilizes the dimer itself, it offers no further stabilization of the VLP, showing that the stability of the dimer is apparently not the limiting factor in VLP stability. PP7 capsids readily assemble *in vitro *in a reaction that requires RNA, raising the prospect that the interior composition of the VLP can be manipulated by specific encapsidation of foreign substances coupled to the RNA.

## Methods

### Proteins and recombinant DNA

The cloning, over-expression and purification of PP7 coat protein have been described in detail elsewhere. To construct the single-chain PP7 dimer, the coat sequence was amplified from pP7CT with Pfu DNA polymerase and a 3'-primer complementary to plasmid vector sequences and a 5'-primer having the sequence: 5'-CCCCCGCCGTTATGGGCAAAACCATCGTTCTTTCGGTC-3'. This introduced a *Bgl *I site near the 5'-end of what would be the downstream copy of the coat protein coding sequence. This was subsequently joined to a naturally occurring *Bgl *I site near the 3'-end of the upstream copy in pP7CT to create the junction sequence shown in Figure 3. The now duplicated sequence was cloned between *Xba *I and *Bam *HI in pET3d for over-expression in *E. coli*. These manipulations resulted in duplication and translational fusion of the two sequences, with the last amino acid of the upstream copy (arginine) joined to the second amino acid (serine) of the downstream copy through a two-amino acid linker (tyr-gly).

### Assay for thermal stability

The thermal stabilities of virus-like particles under various conditions were determined by two methods. In the first a "melting profile" was produced by heating 25 ul samples of PP7 virus-like particles at a concentration of 1.0 mg/ml in 50 mM Tris-HCl, pH 8.5, 100 mM NaCl for 2 min. at specific temperatures. When a reaction contained DTT, it was present at a concentration of 10 mM. At the end of the incubation period, the samples were chilled on ice and then and subjected to centrifugation at 13,000 rpm in an IEC MicroMax microcentrifuge for 5 minutes. The supernatants of these samples, containing the portion of the protein that remained soluble after heat treatment, were removed to a new tube. The insoluble proteins in the pellet were redissolved in 6 M urea. Measurements of the relative quantities of soluble and insoluble protein were performed by Bradford assay [24]. Standard curves were produced using hen lysozyme as a standard and were linear over the range of the assay. For measurement of the quantity of capsids remaining after heat treatment, soluble protein was applied to a 1% agarose gel in 40 mM Tris-acetate, pH 8.0, 2 mM EDTA, and subjected to electrophoresis. The gel was then stained with ethidium bromide and photographed under UV illumination to visualize the RNA-containing VLPs. Protein was stained with coomassie brilliant blue R250. The gel was scanned with a densitometer and the quantity of protein in individual bands was determined by comparison to a standard curve produced by applying dilutions of a known quantity of PP7 virus-like particles to the same gel. The standard curve was linear over the range employed in the assay.

The rates of denaturation were determined by incubation of proteins in 50 mM Tris-HCl, pH 8.5, 100 mM NaCl, with or without DTT at 10 mM at specified temperatures. At time points reactions were quenched on ice and then analyzed for their content of capsids and of soluble and insoluble protein as described above.

### Purification of dimers

Ten milligrams of PP7 or 2PP7 VLPs purified as described previously were incubated for 60 minutes in 1 ml of 50 mM Tris-HCl, pH 8.5, 6 M urea, 10 mM DTT on ice. The resulting protein was dialyzed against 10 mM acetic acid, 50 mM NaCl (about pH 4) and then applied to a 0.9 × 45 cm column of Sephadex G75 and eluted in the same buffer. Fractions of 0.7 ml were collected. Two peaks appeared in the chromatogram. Agarose gel electrophoresis shows that the first peak is made up of VLPs that failed to disassemble. The other, eluting at fraction 20, apparently represents coat protein dimers. In a separate experiment bovine serum albumin (MW = 68,000), ovalbumin (MW = 45,000), chymotrypsinogen (MW = 25,700) and lysozyme (MW = 14,400) were applied to the column as molecular weight standards. The standard proteins yielded a linear plot of elution position versus log molecular weight. Note that BSA was omitted from this analysis because it eluted in or near the void volume. Comparison to the elution behavior of the standards indicates that the second coat protein peak has a molecular weight of about 32,000, a size roughly consistent with the predicted molecular weight of about 28,000 for the coat protein dimer. Protein from the peak fractions was used in the *in vitro *assembly reactions.

### *In vitro *VLP assembly

Purified dimeric PP7 coat protein (0.1 nmol) was added to reactions containing 50 mM Tris-HCl, pH 8.5 and yeast tRNA, MS2 translational operator, or PP7 translational operator RNA in amounts varying by two-fold serial dilution from 0.1 nmol to 6.3 pmol. After 30 minutes, glycerol and bromophenol blue were added and the reactions were subjected to electrophoresis in a 1% agarose gel. RNA was visualized by staining the gels with ethidium bromide followed by photography on a UV transilluminator. MS2 and PP7 translational operator RNAs were produced by transcription *in vitro *as described previously [26]. In some cases RNAs were synthesized in the presence of a ^32^P-labeled nucleotide and could be visualized and quantitated after exposure of the gel to a Packard Cyclone phosphorimager screen. To visualize proteins, gels were stained with coomassie brilliant blue R250.

## List of abbreviations

DTT : dithiothreotol;

EDTA : ethylenediaminetetraacetic acid;

VLP : virus-like particle;  

## Competing interests

The author(s) declare that they have no competing interests.

## Authors' contributions

JCC performed the denaturation experiments. DSP performed all recombinant DNA manipulations, purified coat protein dimers, and conducted the *in vitro *assembly reactions. Both authors read and approved the manuscript.
